# Tetracarpidium conophorum (Mull.Arg) Hutch & Dalziel inhibits FeSO_4_ -induced lipid peroxidation in rat’s genitals

**DOI:** 10.1186/s12906-015-0547-1

**Published:** 2015-03-12

**Authors:** Seun F Akomolafe, Ganiyu Oboh, Afolabi A Akindahunsi, Anthony J Afolayan

**Affiliations:** Department of Biochemistry, Ekiti State University, P.M.B 5363 Ado Ekiti, Nigeria; Department of Biochemistry, Federal University of Technology, Akure, Nigeria; Department of Botany, University of Fort Hare, Alice, South Africa

**Keywords:** Pro-oxidant, Phenolic constituents, Antioxidant activities, Malondialdehyde, Testis

## Abstract

**Background:**

To search for new sources of safe and inexpensive antioxidant agents which can be used to treat various oxidative stress - related diseases, the phenolic contents of leaf of *Tetracarpidium conophorum* were characterized and its effect on pro-oxidant induced oxidative stress in rat’s genitals for the first time was investigated.

**Methods:**

The aqueous extract of the plant was prepared, the antioxidant activities of the extract were then evaluated using spectrophotometric method.

**Results:**

The result revealed that the introduction of aqueous extract of the plant caused significant concentration-dependent decrease (P < 0.05) in the MDA content of the Fe^2+^ -stressed testes and penis homogenates. The least MDA production occurred at the highest concentration of the extract (0.625 mg/mL). However, characterization of the extract with HPLC revealed that its major constituents were gallic acid, catechin, chlorogenic acid, caffeic acid, coumarin, rutin, quercitrin, quercetin, kaempferol and luteolin. Also, the result revealed that the ABTS* scavenging ability of the extract was 4.60 mmol/100 g while its vitamin C content was 23.49 mg/g which indicated that the plant is very rich in vitamin C. Furthermore, the extract scavenged DPPH, NO, OH* radicals and chelated Fe^2+^ in a dose-dependent manner.

**Conclusion:**

The inhibitory effect of *Tetracarpidium conophorum* leaves could be attributed to the high levels of quercitrin, quercetin and luteolin and the mechanism through which these compounds possibly do this, could be by their radical scavenging abilities.

## Background

Infertility is a major clinical problem, affecting people medically and psychosocially. It has been established that 15% of all couples in the United States are infertile, and that the male factor is responsible for 25% of these cases [1]. In Nigeria, this problem affects one out of five men. Oxidative stress (OS) has been identified as one of the major factors that affects fertility status and its role in male infertility has been well documented in recent years. Oxygen is essential to sustain life, at physiological levels, reactive oxygen species (ROS) are necessary to maintain normal cell functions [2]. Conversely, the breakdown products of oxygen such as ROS can be detrimental to cell function and survival [3].

Although iron is necessary physiologically as component of many enzymes and proteins, free iron in the cytosol and mitochondria could cause considerable oxidative damage by acting catalytically in the production of ROS which have the potential to damage cellular lipids, nucleic acids, proteins and carbohydrate resulting in wide ranging impairment in cellular function and integrity [4].

The mechanism by which iron causes this deleterious effect is that Fe (II) reacts with hydrogen peroxide (H_2_O_2_) to produce the hydroxyl radical (OH) via the Fenton reaction, whereas superoxide can react with iron (III) to regenerate iron (II) that participates in the Fenton reaction [5]. The overproduction of ROS can lead to direct attack on the polyunsaturated fatty acids of the cell membranes and induce lipid peroxidation. Spermatozoa, like any other aerobic cells, are constantly facing the oxygen-paradox [6] because they are rich in polyunsaturated fatty acids (PUFA) and thus, they are very susceptible to ROS attack which results in decrease sperm motility [7].

Malondialdehyde (MDA) is the end product of lipid peroxidation. It is a process where ROS degrade polyunsaturated fatty acids. MDA is a reactive aldehyde and is one of the many reactive electrophile species that cause toxic stress in cells. The production of this aldehyde is used as a biomarker to measure the level of oxidative stress in an organism [8].

However, the most likely and practical way to fight degenerative diseases is to improve body antioxidant status, which could be achieved by higher consumption of fruits and vegetables. Phenolic compounds are important group of secondary metabolites which are synthesized by plants.

*Tetracarpidium conophorum* (Mull.Arg) Hutch & Dalziel commonly called African walnut is a climbing shrub in the family Euphorbiaceae. The plant is locally cultivated mainly for the nuts which are cooked and consumed as snacks [9]. It is locally used by the elderly people for the treatment of constipation. The amino and fatty acids components of the nut are used for the treatment of prolonged and constant hiccups [10]. The barks are used in coffee as laxative and also chewed to reduce toothache. The leaves, bark and fruit of the plant are used medicinally and their uses include giddiness, toothache, eczema, pruritus, psoriasis, common cold and prostate cancer [11]. Also, in West Africa the leaves are used as male fertility agent and in the treatment of dysentery. *T. conophoum* seed have been shown to have signifcant effect (P ≤ 0.05) on testosterone and estradiol levels, sperm motility (progresive motile sperm and non progresive motile sperm), testes and epididymides weight of the rats treated with various doses of the seed powder while it increased the level of LH, FSH, sperm viability and sperm count [12,13].

Although a lot have been reported on the chemical characterization of the phytoconstituents and antioxidant properties of *T. conophorum* seeds, there is still limited information on the potential of its leaves in the management and prevention of infertility diseases associated with oxidative stress. Hence, the objective of this study is to investigate the inhibitory effect of aqueous extract of *T. conophorum* leaves on FeSO_4_ -induced lipid peroxidation in rat testes and penis *in vitro*.

## Methods

### Collection and identification of plant samples

Fresh samples of *T. conophorum leaves* were obtained from a farm land near Akure metropolis, Nigeria. Authentication of the sample was carried out at the Department of Plant Science, Ekiti State University, Ado-Ekiti where voucher specimen (number UHAE 335) was deposited at the herbarium of the same Department.

### Experimental animals

Ten male Wistar albino rats weighing between 190 g and 250 g were purchased from the Central Animal House, Department of Biochemistry, University of Ilorin, Nigeria. They were housed in stainless steel cages under controlled conditions of a 12-hour light/dark cycle and 28°C temperature. The rats were allowed asses to food and water *ad libitum*. The cages were cleaned once daily. This study was carried out following approval from the ethical committee on the use and care of experimental animals of the Department of Biochemistry, Federal University of Technology, Akure, Nigeria. The research also adhered strictly to the Principles of Laboratory Animal Care (NIH Publication, No. 85–23).

### Chemicals and reagents

Chemicals and reagents used such as thiobarbituric acid (TBA), 1,10-phenanthroline, deoxyribose, acetonitrile, acetic, gallic, chlorogenic and caffeic acids were purchased from Merck (Darmstadt, Germany). Catechin, coumarin, quercetin, quercitrin, rutin, luteolin and kaempferol were acquired from Sigma Chemical Co. (St. Louis, MO, USA). Folin-Ciocalteau’s reagent were procured from Sigma-Aldrich, Inc. (St. Louis, MO). Trichloroacetic acid (TCA) and MDA were sourced from Sigma-Aldrich, Chemie GmbH (Steinheim, Germany). Hydrogen peroxide, methanol, acetic acid, and HCl were sourced from BDH Chemicals Ltd. (Poole, England). Sodium carbonate, AlCl_3_, potassium acetate, Tris–HCl buffer, sodium dodecyl sulphate (SDS), FeSO_4_, potassium ferricyanide and ferric chloride were of analytical grade. A UV-visible spectrophotometer (Model 6305; Jenway, Barlo world Scientific, Dunmow, United Kingdom) was used to measure absorbance. High performance liquid chromatography (HPLC-DAD) was performed with a Shimadzu Prominence Auto Sampler (SIL-20A) HPLC system (Shimadzu, Kyoto, Japan), equipped with Shimadzu LC-20AT reciprocating pumps connected to a DGU 20A5 degasser with a CBM 20A integrator, SPD-M20A diode array detector and LC solution 1.22 SP1 software.

### Aqueous extract preparation

The fresh leaf samples were air dried, homogenized and kept in an air-tight container prior to the extraction. The powdered sample (1 g) was soaked in 100 mL of distilled water for 24 hours [14]. The mixture was later filtered and the filtrate centrifuged at 400 g for 10 minutes. The clear supernatant was used for subsequent analysis.

### 2,2-azinobis(3-ethylbenzo-thiazoline-6-sulfonate) ABTS^*^ scavenging ability

The ABTS* scavenging ability of the extract was determined according to the method of Re *et al*. [15] ABTS* was generated by reacting an ABTS aqueous solution (7 mmol L^−1^) with K_2_S_2_O_8_ (2.45 mmol L^−1^) in the dark for 16 hours and adjusting the absorbance with ethanol to 734–0.700 nm with ethanol. Then, 0.2 ml of the extract was added to 2.0 ml ABTS* solution and the absorbance was measured at 734 nm after 15 minutes. The result was calculated and reported as trolox equivalent antioxidant capacity.

### Preparation of tissue homogenate

The rats were decapitated under mild diethyl ether anesthesia and the testes and penis tissues were rapidly dissected and placed on ice and weighed. This tissue was separately homogenized in cold saline (1:10) with about 10-up-and-down strokes at approximately 1200 rev/minutes in a Teflon-glass homogenizer. The homogenate was centrifuged for 10 minutes at 3000 *g* to yield a pellet that was discarded, and a low-speed supernatant (S1) containing mainly water, proteins and lipids was kept for the lipid peroxidation assay [16].

### Lipid peroxidation and thiobarbibutric acid reactions

The lipid peroxidation assay was carried out using the method of Ohkawa *et al*. [17] Briefly, 100 μL S1 fraction was mixed with a reaction mixture containing 30 μL of 0.1 M Tris- HCl buffer (pH 7.4), aqueous extract of *T. conophorum* leaves (0–100 μL) and 30 μL of 250 mM freshly prepared FeSO_4_. The volume was made up to 300 μL with water before incubation at 37°C for 1 hour. The color reaction was developed by adding 300 μL of 8.1% SDS to the reaction mixture containing S1. This was subsequently followed by the addition of 500 μL of acetic acid/HCl (pH 3.4) and 500 μL 0.8% TBA. The mixture was incubated at 100°C for 1 hour. The absorbance of thiobarbituric acid reactive species produced was measured at 532 nm. MDA produced was expressed in percentage (%). The EC_50_ (extract concentration required to inhibit 50% of MDA produced) value were calculated using non-linear regression analysis.

### DPPH free radical scavenging ability

The free radical scavenging ability of the extract against DPPH (1,1-diphenyl-2 picrylhydrazyl) free radical was evaluated as described by Gyamfi *et al*. [18] Briefly, an appropriate dilution of the extracts (1 mL) was mixed with 1 mL of 0.4 mM methanolic solution containing DPPH radicals, the mixture was left in the dark for 30 minutes and the absorbance was measured at 516 nm. The control was carried out using 2 mL DPPH solution without the test samples. The DPPH free radical scavenging ability was subsequently calculated as follow: DPPH scavenging ability (%) = [(absorbance of control − absorbance of samples)/absorbance of control] × 100.

### Degradation of deoxyribose (Fenton’s reaction)

The ability of the extract to prevent Fe^2+^/H_2_O_2_- induced decomposition of deoxyribose was carried out using the method of Halliwell and Gutteridge [19]. Briefly, freshly prepared aqueous extract (0–100 μL) was added to a reaction mixture containing 120 μL 20 mM deoxyribose, 400 μL 0.1 M phosphate buffer, 40 μL 20 mM hydrogen peroxide and 40 μL 500 mM FeSO4, and the volume made to 800 μL with distilled water. The reaction mixture was incubated at 37°C for 30 minutes, and the reaction was stopped by the addition of 500 μL of 2.8% TCA, this was followed by the addition of 400 μL of 0.6% TBA solution. The tubes were subsequently incubated in boiling water for 20 minutes. The absorbance was measured at 532 nm and the percentage hydroxyl radical scavenging ability by the extract was calculated using the following equation:

$$ \left[\left({\mathrm{Abs}}_{\mathrm{ref}} - {\mathrm{Abs}}_{\mathrm{sample}}\right)/{\mathrm{Abs}}_{\mathrm{ref}}\right] \times 100 $$

where Abs_ref_ = absorbance of the reference (reacting mixture without the test sample) and Abs_sample_ = absorbance of reacting mixture with the test sample.

### Nitric oxide scavenging assay

The nitric oxide scavenging activity of the extract was evaluated by the method of Igbinosa *et al*. [20] 25 mM sodium nitroprusside (1 ml) prepared in 0.5 mM phosphate buffer saline (pH 7.4) was added to (100–400 μL) of plant extract and vortexed. The mixture was incubated for 2 hours at 37°C and thereafter, 1 ml of the mixture was taken and mixed with 1 ml of Griess reagent (equal volumes of 1% sulphanilic acid prepared in 2% orthophosphoric acid and 0.01% (w/v) naphthylenediamine dichloride) and incubated at room temperature for 30 minutes. The absorbance was read at 546 nm and the percentage nitric oxide inhibition by the extract was calculated using the following equation:

$$ \mathrm{NO}\ \mathrm{scavenging}\ \mathrm{activity}\ \left(\%\right) = \left[\left(\mathrm{Abs}\ \mathrm{control}\ \hbox{--}\ \mathrm{Abs}\ \mathrm{sample}\right)\right]/\left(\mathrm{Abs}\ \mathrm{control}\right) \times 100 $$

Where Abs control is the absorbance of NO radicals; Abs sample is the absorbance of NO radical + sample or standard.

### Fe^2+^ chelation assay

The Fe^2+^ chelating ability of the extract was determined using a modified method of Untel *et al*. [21] Freshly prepared 500 mM FeSO4 (150 μL) was added to a reaction mixture containing 168 μL of 0.1 M Tris–HCl (pH 7.4), 218 μL saline and the extracts (100–400 μL). The reaction mixture was incubated for 5 minutes, before the addition of 13 μL of 0.25% 1, 10-phenanthroline. The absorbance was subsequently measured at 510 nm in a spectrophotometer. The Fe (II) chelating ability was subsequently calculated with respect to the reference which contains all the reagents without the test sample.

### Quantification of compounds by HPLC-DAD

Reverse phase chromatographic analyses were carried out under gradient conditions using C_18_ column (4.6 mm × 150 mm) packed with 5 μm diameter particles; the mobile phase was water containing 2% formic acid (A) and acetonitrile (B), and the composition gradient was: 17% of B until 10 minutes and changed to obtain 20%, 30%, 50%, 60%, 70%, 20% and10% B at 20, 30, 40, 50, 60, 70 and 80 minutes, respectively, following the method described by Kamdem *et al*. [22] with slight modifications. *Tetracarpidium conophorum* aqueous extract was analyzed at a concentration of 20 mg/mL. The presence of ten antioxidants compounds was investigated, namely, gallic acid, chlorogenic acid, caffeic acid, catechin, coumarin, quercetin, quercitrin, rutin, luteolin and kaempferol. Identification of these compounds was performed by comparing their retention time and UV absorption spectrum with those of the commercial standards. The flow rate was 0.7 mL/min, injection volume 40 μl and the wavelength were 270 nm for gallic acid, 276 nm for coumarin, 280 nm for catechin, 327 nm for caffeic and chlorogenic acids, and 365 nm for quercetin, quercitrin, rutin, kaempferol and luteolin. The samples and mobile phase were filtered through 0.45 μm membrane filter (Millipore) and then degassed by ultrasonic bath prior to use. Stock solutions of standards references were prepared in the HPLC mobile phase at a concentration range of 0.025 – 0.250 mg/ml for catechin, coumarin, quercetin, quercitrin, rutin, kaempferol and luteolin; and 0.030 – 0.350 mg/ml for caffeic, chlorogenic and gallic acids. The chromatography peaks were confirmed by comparing its retention time with those of reference standards and by DAD spectra (200 to 600 nm). Calibration curve for catechin: Y = 13649x + 1257.8 (r = 0.9995); coumarin: Y = 11974x + 1309.5 (r = 0.9999); gallic acid: Y = 12890x + 1253.7 (r = 0.9998); caffeic acid: Y = 13078x + 1186.3 (r = 0.9991); chlorogenic acid: Y = 11952x + 1187.5 (r = 0.9996); rutin: Y = 12657x + 1238.9 (r = 0.9999); quercetin: Y = 13591x + 1318.7 (r = 0.9998); quercitrin: Y = 11783x + 1263.8 (r = 0.9994); kaempferol: Y = 11974x + 1271.0 (r = 0.9999) and luteolin Y = 13528x + 1367.2 (r = 0.9997). All chromatography operations were carried out at ambient temperature and in triplicate. The limit of detection (LOD) and limit of quantification (LOQ) were calculated based on the standard deviation of the responses and the slope using three independent analytical curves, as defined by Kamdem *et al*. [22] LOD and LOQ were calculated as 3.3 and 10 σ/S, respectively, where σ is the standard deviation of the response and S is the slope of the calibration curve.

### Data analysis

The results of the replicates were pooled and expressed as mean ± standard deviation. Analysis of variance (ANOVA), Tukey test, and student *t*-test were carried out where appropriate to analyze the result. Significance was accepted at P ≤ 0*.*05.

## Results and discussion

**Figure 1:**
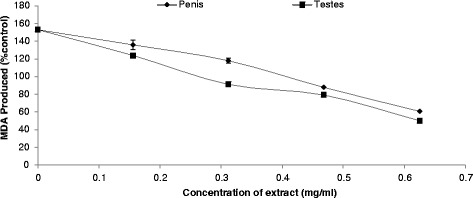
**Inhibition of Fe** ^**2+**^ **-induced lipid peroxidation in rat’s testes and penis by aqueous extract of** ***T. conophorum*** **leaves.**

One of the major mechanisms of cell injury in aerobic organisms subjected to oxidative stress is lipid peroxidation of biological membranes [23]. Incubation of the rat’s testes and penis homogenates in the presence of Fe^2+^ (a transition metal) caused a significant increase in the MDA content of the testes and penis (Figure 1). These findings agree with our earlier reports on the interaction of Fe^2+^ with the testes, in which Fe^2+^ was shown to be a very potent initiator of lipid peroxidation in the testes [24]. This result run partially in parallel with the pervious study by Akintunde *et al*. [25] who recorded elevated malondialdehyde (MDA) level in rat testes exposed to chronic ingestion of leachate samples which contained elevated level of mixed-metals over the permissible levels. The increased lipid peroxidation in the presence of Fe^2+^ could be attributed to the fact that Fe^2+^ can catalyze one-electron transfer reactions that generate ROS such as reactive hydroxyl radical (^•^OH) which is formed from H_2_O_2_ through the Fenton reaction. Iron also decomposes lipid peroxides, thus generating peroxyl and alkoxyl radicals, which favors the propagation of lipid peroxidation [5].

However, the introduction of aqueous extract of *T. conophorum* leaves caused a significant concentration-dependent decrease in the MDA content of the Fe^2+^-stressed testes homogenate from 123.88% down to 50.11% and penis homogenate from 135.87% down to 60.72% with the least MDA production occurring at the introduction of the highest concentration of the extract (0.625 mg/mL) (Figure 1). The mode of inhibition of Fe^2+^ - induced lipid peroxidation could be because of the possibility of the water-extractable phytochemicals to form complexes with the Fe^2+^ thereby preventing them from catalyzing the initiation of lipid peroxidation, or the phytochemicals could have mooped up the free radicals produced by the Fe^2+^ - catalyzed reaction [14]. Judging from the EC_50_ value, the concentration of the *T. conophorum* leaf extract that will cause 50% inhibitory effect on FeSO_4_ –induced lipid peroxidation in testes is 0.43 mg/ml while in penis is 0.72 mg/ml (Table 1).

**Table 1:** **Antioxidant capacity, vitamin C content and EC**_**50**_ **values of inhibition of FeSO**_**4**_ **-induced lipid peroxidation in rat’s testes and penis by aqueous extract of** ***T. conophorum*** **leaves**

**Antioxidant capacity(mmol. TEAC/100 g)**	**Vitamin C content(mg/g)**	**EC**_**50**_ **value in testes(mg/ml)**	**EC**_**50**_ **value in penis(mg/ml)**
4.60 ± 0.23	23.49 ± 0.04	0.43 ± 0.12	0.72 ± 0.38

Values represent mean ± standard deviation, number of samples *n* = 3.

**Figure 2:**
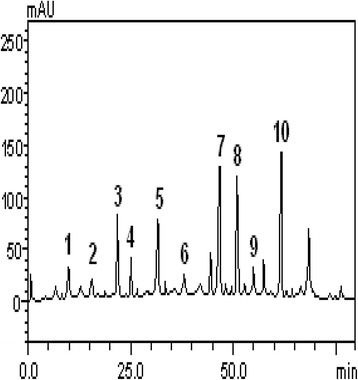
**Representative high performance liquid chromatography profile of** ***Tetracarpidium conophorum*** **aqueous extract.** Gallic acid (peak 1), catechin (peak 2), chlorogenic acid (peak 3), caffeic acid (peak 4), coumarin (peak 5), rutin (peak 6), quercitrin (peak 7), quercetin (peak 8), kaempferol (peak 9) and luteolin (peak 10).

The antioxidant properties of plants have been linked to the presence of an array of important phenolic and non-phenolic phytochemicals including phenolic acids, flavonoids and alkaloids [26]. However, characterization of the extract with HPLC revealed that the major constituent of the aqueous extract of *T. conophorum* leaves are gallic acid, catechin, chlorogenic acid, caffeic acid, coumarin, rutin, quercitrin, quercetin, kaempferol and luteolin of which the level of quercitrin, quercetin and luteolin were very high (Table 2, Figure 2). Phenolic compounds are strong antioxidants capable of removing free radicals, chelate metal catalysts, activate antioxidant enzymes, reduce α-tocopherol radicals and inhibit oxidases [26]. They can protect the human body from free radicals whose formation is associated with the normal metabolism of aerobic cells. Therefore, the protection of testes and penis tissue from Fe^2+^ -induced lipid peroxidation by the aqueous extract of *Tetracarpidium conophorum* leaves could be attributed to very high level of quercitrin, quercetin and luteolin. These flavonoids (Quercetin, quercetrin and luteolin) have been reported to be powerful antioxidants [27] whose effect have been linked to their two aromatic rings each containing at least one hydroxyl, which are connected through a three-carbon bridge and become part of a six-member heterocyclic ring thereby making them acting as chain breaking antioxidants which scavenge reactive oxygen species [28-30].

**Table 2:** **Components of** ***Tetracarpidium conophorum*** **aqueous leaf extract**

**Compounds**	***Tetracarpidium conophorum***
	**mg/g**
Gallic acid	2.47 ± 0.03a
Catechin	1.93 ± 0.01b
Chlorogenic acid	6.71 ± 0.01c
Caffeic acid	3.85 ± 0.02d
Coumarin	6.79 ± 0.03c
Rutin	1.90 ± 0.01b
Quercitrin	10.47 ± 0.03e
Quercetin	10.28 ± 0.01e
Kempferol	1.89 ± 0.02b
Luteolin	12.56 ± 0.01f

Results are expressed as mean ± standard deviations (SD) of three determinations. Values followed by different letters differ by Tukey test at p < 0.05.

Antioxidants carry out their protective role on cells either by preventing the production of free radicals or by neutralizing or scavenging free radicals produced in the body or by reducing or chelating the transition metal composition of foods [31,22]. In an attempt to explain the main mechanism through which the aqueous extract of the plant protect testes tissue against Fe^2+^ induced lipid peroxidation, the DPPH radical scavenging, ABTS radical scavenging and Fe^2+^ chelating abilities were assessed.

ABTS, a blue green chromophore is mostly reactive toward phenolics, thiols and other antioxidants [32]. The ABTS* free radical scavenging ability model has the advantage of being more versatile due to the minimal spectral interference as the absorption maximum used is 760 nm, a wavelength not normally encountered with natural products [33]. The result revealed that the ABTS* scavenging ability of the aqueous extract is 4.60 mmol/100 g while its vitamin C content is 23.49 mg/g which indicate that the plant is very rich in vitamin C (Table 1). The ABTS* free radical scavenging ability of the extract could be because of the hydrogen donating ability of the polyphenolics present in the extract.

**Figure 3:**
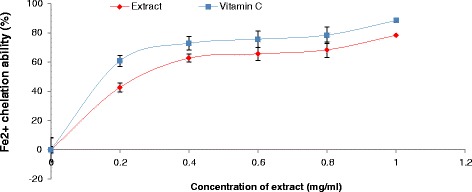
**DPPH radical scavenging ability of aqueous extract of** ***T. conophorum*** **leaves and vitamin C.**

**Figure 4:**
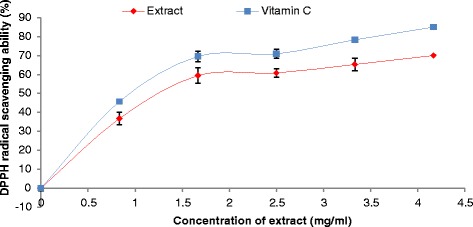
**Fe** ^**2+**^ **chelating ability of aqueous extract of** ***T. conophorum*** **leaves and vitamin C.**

The prevention of the chain initiation step by scavenging various reactive species such as free radicals is considered to be an important antioxidant mode of action [34]. DPPH is a free radical donor that accepts an electron or hydrogen to become a stable diamagnetic molecule [35]. The tendencies of electron or hydrogen donation are critical factors in characterizing antioxidant activity that involves free radical scavenging [36]. Our findings revealed that the extract scavenged DPPH radicals in a dose-dependent manner (Figure 3). Also, the plant aqueous extract chelate Fe^2+^ at all the concentrations used (Figure 4), nevertheless vitamin C chelate Fe^2+^ and scavenge DPPH radical better than the *T. conophorum* extract.

**Figure 5:**
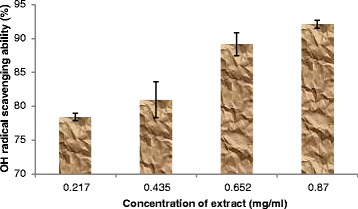
**OH radical scavenging ability of aqueous extract of** ***T. conophorum*** **leaves.**

Furthermore, the results revealed that the extract was able to scavenge OH* radical produced from the decomposition of deoxyribose in Fenton reaction in a concentration dependent manner (Figure 5). This observation suggests that the extract can be used as an alternative remedy to synthetic antioxidants in combating the oxidative activity of hydroxyl radical.

**Figure 6:**
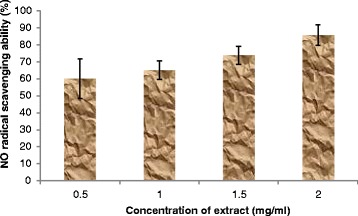
**Nitric oxide radical scavenging ability of aqueous extract of** ***T. conophorum*** **leaves.**

Nitric oxide (NO) radical is generated from sodium nitroprusside at physiological pH. It has the ability of changing the structural and functional behavior of many cellular components because it is a highly reactive compound [37]. The aqueous extract of *T. conophorum* leaves inhibited NO radical in a concentration dependent manner (Figure 6). This inhibitory effect of the extract against NO radical can be attributed to its ability to compete with oxygen and its derivatives [38]. Our results were in agreement with a similar work done by Amaeze *et al*. [39] who reported that ethanol-water extract of *T. conophorum* leaves had the highest ferric reducing property and antioxidant activity on DPPH and NO radicals when compared with methanolic extract.

## Conclusion

The protection of testes and penis tissues from Fe^2+^ -induced lipid peroxidation by the aqueous extract of *Tetracarpidium conophorum* leaves could be attributed to very high level of quercitrin, quercetin and luteolin and the mechanism through which they possibly do this could be by their radical scavenging abilities.

## References

[1] Sharlip ID, Jarow JP, Belker AM, Lipshultz LI, Sigman M, Thomas AJ (2002). Best practice policies for male infertility. Fertil Steril.

[2] Badade ZG, Samant PM (2011). Role of oxidative stress in male infertility. J Bio Sci Res.

[3] de Lamirande E, Gagnon C (1995). Impact of reactive oxygen species on spermatozoa: a balancing act between beneficial and detrimental effects. Hum Reprod.

[4] Britton RS, Leicester KL, Bacon BR (2002). Iron toxicity and chelation therapy. Int J Hematol.

[5] Zago MP, Verstraeten SV, Oteiza PI (2000). Zinc in the prevention of Fe2+ initiated lipid and protein oxidation. Biol Res.

[6] Sies H (1993). Strategies of antioxidant defense. Eur J Biochem.

[7] Aitken J, Krausz C, Buckingham D (1994). Relationships between biochemical markers for residual sperm cyto-plasm, reactive oxygen species generation, and the presence of leukocytes and precursor germ cells in human sperm suspensions. Mol Reprod Dev.

[8] Murray RK, Granner DK, Mayes PA, Rodwell VW (2000). Harper’s biochemistry.

[9] Oke OL (1995). Leaf protein research in Nigeria Ibadan.

[10] Oladiji AT, Abodunrin TP, Yakubu MT (2007). Some physicochemical characteristics of the oil from Tetracarpidium conophorum (Mull. Arg.) Hutch. Dalz nut. *International*. J Biochem Mol Biol.

[11] Odugbemi O, Akinsulire O, Odugbemi T (2008). Medicinal plants by species names. Outlines and pictures of medicinal plants from Nigeria.

[12] Enujiugha VN (2003). Chemical and functional characteristics of conophor nut. Pak J Nutr.

[13] Ikpemel EV, Ekaluo UB, Udensi O, Ekerete EE, Ekpo PB, Asuquo BO (2014). Sperm quality and hormone profile of male Albino rats FED with seds of African Walnut (Tetracarpidium conophorum, Mul). An Res Rev Biol.

[14] Oboh G, Puntel RL, Rocha JBT (2007). Hot pepper (Capsicum annuum, tepin and Capsicum chinese, Habanero) prevents Fe^2+^ -induced lipid peroxidation in brain –*in vitro*. Food Chem.

[15] Re R, Pellegrini N, Proteggente A, Pannala A, Yang M, Rice-Evans C (1999). Antioxidant activity applying an improved ABTS radical cation decolorisation assay. Free Radic Biol Med.

[16] Belle NAV, Dalmolin GD, Fonini G (2004). Polyamines reduces lipid peroxidation induced by different pro-oxidant agents. Brain Res.

[17] Ohkawa H, Ohishi N, Yagi K (1979). Assay for lipid peroxides in animal tissues by thiobarbituric acid reaction. Anal Biochem.

[18] Gyamfi MA, Yonamine M, Aniya Y (1999). Free-radical scavenging action of medicinal herbs from Ghana: Thonningia Sanguinea on experimentally-induced liver injuries. Gen Pharmacol.

[19] Halliwell B, Gutteridge JMC (1981). Fomation of a thiobarbituric-acidreactive substance from deoxyribose in the presence of iron salts: the role of superoxide and hydroxyl radicals. FEBS Lett.

[20] Igbinosa OO, Igbinosa IH, Chigor VN, Uzunuigbe OE, Oyedemi SO, Odjadjare EE (2011). Polyphenolic contents and antioxidant potential of stem bark extracts from *Jatropha curcas* (Linn). Int J Mol Sci.

[21] Puntel PRL, Nogueira CW, Rocha JBT (2005). Krebs cycle intermediates modulate Thiobarbituric Reactive Species (TBARS) production in rat brain *in Vitro*. Neuro Chem Res.

[22] Kamdem JP, Olalekan EO, Hassan W, Kade J, Yetunde O, Boligon AA (2013). *Trichilia catigua* (Catuaba) bark extract exerts neuroprotection against oxidative stress induced by different neurotoxic agents in rat hippocampal slices. Ind Crop Prod.

[23] Oboh G, Rocha JBT (2007). Antioxidant in foods: a new challenge for food processors: leading edge antioxidants research.

[24] Akomolafe SF, Oboh G, Akindahunsi AA, Akinyemi AJ, Adeyanju O (2012). Inhibitory effect of aqueous extract of *Moringa oleifera* and *Newbuoldia laevis* leaves on ferrous sulphate and sodium nitroprusside induced oxidative stress in rat’s testes *in vitro*. Open J Med Chem.

[25] Akintunde JK, Oboh G, Akindahunsi AA (2013). Testicular membrane lipid damage by complex mixture of leachate from municipal battery recycling site as indication of idiopathic male infertility in rat. Interdiscip Toxicol.

[26] Cheplick S, Kwon Y, Bhowmik P, Shetty K (2007). Clonal variation in raspberry fruit phenolics and relevance for diabetes and hypertension management. J Food Biochem.

[27] Vinson AJ, Hontz B (1995). Phenol antioxidant index: comparative antioxidant effectiveness of red and white wines. J Agric Food Chem.

[28] Yukiko H, Ogawa S, Fukui S (1994). The correlation between active oxygen scavenging and antioxidants of flavonoids. Free Rad Biol Med.

[29] Robak J, Gryglewski RJ (1988). Flavonoids are scavengers of superoxide anions. Biochem Pharmacol.

[30] Husain S, Cillard J, Cillard P (1987). Hydroxy radical scavenging activity of flavonoids. Phytochemistry.

[31] Amic D, Davidovic-Amic D, Beslo D, Trinajstic N (2003). Structureradical scavenging activity relationship of flavonoids. Croat Chem Acta.

[32] Alia M, Horcajo C, Bravo L, Goya L (2003). Effect of grape antioxidant dietary fiber on the total antioxidant capacity and the activity of liver antioxidant enzymes in rats. Nutr Res.

[33] Walker RB, Everette JO (2009). Comparative reaction rates of various antioxidants with ABTS 430 radical cation. J Agric Food Chem.

[34] Dastmalchi K, Dorman HJD, Kosar M, Hil-tunen R (2007). “Chemical composition and in vitro antioxidant evaluation of a water soluble Moldavian Balm (Draco-cephalum moldavica L.) extract”, LWT—food. Sci Technol.

[35] Je JY, Park PJ, Kim EK, Ahn CB (2009). Antioxidant and Angiotensin I converting enzyme inhibitory activity of *Bambusae caulis* in Liquamen. Food Chem.

[36] Hu C, Zhang Y, Kitts DD (2000). Evaluation of antioxidant and prooxidant activities of bamboo *Phyl-lostachys niger* var. Henonis leaf extract *in Vitro*. J Agric Food Chem.

[37] Ashokkumar D, Thamilselvan V, Senthilkumar GP, Mazumder UK, Gupta M (2008). Antioxidant and free radical scavenging effects of *Lippianodiflora*. Pharm Biol.

[38] Marcocci L, Packer L, Lefaiz DMT, Sekaki A, Albert MG (1994). Antioxidant action of *Ginkgo biloba* extract (Egb 761). Methods Enzymol.

[39] Amaeze OU, Ayoola GA, Sofidiya MO, Adepoju-Bello AA, Adegoke AO, Coker HAB (2011). Evaluation of Antioxidant Activity of *Tetracarpidium conophorum* (Müll. Arg) Hutch & Dalziel Leaves. Oxid Med Cell Longev.

